# Thymic Stromal Lymphopoietin (TSLP) Is Cleaved by Human Mast Cell Tryptase and Chymase

**DOI:** 10.3390/ijms25074049

**Published:** 2024-04-05

**Authors:** Luisa Canè, Remo Poto, Francesco Palestra, Ilaria Iacobucci, Marinella Pirozzi, Seetharaman Parashuraman, Anne Lise Ferrara, Amalia Illiano, Antonello La Rocca, Edoardo Mercadante, Piero Pucci, Gianni Marone, Giuseppe Spadaro, Stefania Loffredo, Maria Monti, Gilda Varricchi

**Affiliations:** 1 Department of Translational Medical Sciences, University of Naples Federico II, 80131 Naples, Italy; canel@ceinge.unina.it (L.C.); remo.poto@gmail.com (R.P.); f.palestra97@gmail.com (F.P.); anneliseferrara@gmail.com (A.L.F.); marone@unina.it (G.M.); spadaro@unina.it (G.S.); stefania.loffredo2@unina.it (S.L.); 2 World Allergy Organization (WAO), Center of Excellence (CoE), 80131 Naples, Italy; 3 CEINGE Advanced Biotechnologies F. Salvatore, 80131 Naples, Italy; ilaria.iacobucci@unina.it (I.I.); pucci@unina.it (P.P.); 4 Department of Chemical Sciences, University of Naples Federico II, 80126 Naples, Italy; 5 Institute of Experimental Endocrinology and Oncology, National Research Council (CNR), 80131 Naples, Italy; m.pirozzi@ieos.cnr.it (M.P.); seetharaman.parashuraman@cnr.it (S.P.); 6 Thoracic Surgery Unit—Istituto Nazionale Tumori IRCCS Fondazione G. Pascale, 80131 Naples, Italy; a.illiano@istitutotumori.na.it (A.I.); a.larocca@istitutotumori.na.it (A.L.R.); edoardo.mercadante@istitutotumori.na.it (E.M.); 7 Center for Basic and Clinical Immunology Research (CISI), University of Naples Federico II, 80131 Naples, Italy

**Keywords:** airway remodeling, asthma, chymase, epithelial cells, macrophage, mast cell, tryptase, TSLP, VEGF-A

## Abstract

Thymic stromal lymphopoietin (TSLP), mainly expressed by epithelial cells, plays a central role in asthma. In humans, TSLP exists in two variants: the long form TSLP (lfTSLP) and a shorter TSLP isoform (sfTSLP). Macrophages (HLMs) and mast cells (HLMCs) are in close proximity in the human lung and play key roles in asthma. We evaluated the early proteolytic effects of tryptase and chymase released by HLMCs on TSLP by mass spectrometry. We also investigated whether TSLP and its fragments generated by these enzymes induce angiogenic factor release from HLMs. Mass spectrometry (MS) allowed the identification of TSLP cleavage sites caused by tryptase and chymase. Recombinant human TSLP treated with recombinant tryptase showed the production of 1-97 and 98-132 fragments. Recombinant chymase treatment of TSLP generated two peptides, 1-36 and 37-132. lfTSLP induced the release of VEGF-A, the most potent angiogenic factor, from HLMs. By contrast, the four TSLP fragments generated by tryptase and chymase failed to activate HLMs. Long-term TSLP incubation with furin generated two peptides devoid of activating property on HLMs. These results unveil an intricate interplay between mast cell-derived proteases and TSLP. These findings have potential relevance in understanding novel aspects of asthma pathobiology.

## 1. Introduction

Bronchial epithelial cells are not only a physical barrier, but also a major component of the immune system and maintain lung tissue homeostasis [1]. Environmental stimuli can damage bronchial epithelial cells, representing a first immunologic event in several inflammatory lung disorders [2,3]. Damaged epithelial cells release several cytokines, termed alarmins [i.e., thymic stromal lymphopoietin (TSLP), IL-33, and IL-25], which drive asthma immunology [4,5]. TSLP, a pleiotropic cytokine initially cloned in a murine thymic stromal cell line [6], is mainly expressed by lung epithelial cells [7,8,9,10,11,12]. TSLP is also expressed by human dendritic cells (DCs) [13], mast cells [7,14,15,16], monocytes [13,17], macrophages [17,18], and granulocytes [19]. This cytokine is also released from structural cells, such as airway smooth muscle cells [20] and fibroblasts [21]. TSLP activates a heteromeric complex composed of a thymic stromal lymphopoietin receptor (TSLPR) chain and interleukin 7 receptor-α (IL-7Rα) [22,23]. The dimerization of both receptor chains upon TSLP binding results in the activation of Janus kinases (JAKs) and signal transducer and activator of transcription 5 (STAT5), which represents a critical downstream biochemical event [24,25].

TSLP has been broadly implicated in the pathogenesis of type 2 inflammatory disease [26,27]. TSLP plays a key role in the initiation of the type 2 immune response through the activation of group 2 innate lymphoid cells (ILC2) [28], T helper 2 (Th2) cells [29], and dendritic cells (DCs) [16,26]. In addition, TSLP activates effector cells in asthma such as human lung macrophages (HLMs) [18], mast cells [14], and eosinophils [30]. The above considerations have led to the conclusion that TSLP is a master orchestrator of asthma pathobiology [5,27] and the approval of a monoclonal antibody anti-TSLP (tezepelumab) highly effective in severe asthma treatment [31,32,33].

Harada and collaborators identified two variants of TSLP in human bronchial epithelial cells: the long form (lfTSLP) and a shorter TSLP isoform (sfTSLP) [25,34,35]. The short form TSLP (sfTSLP) overlaps the C-terminus of the lfTSLP [27]. The lfTSLP has a signal peptide encoded in the first 28 amino acids at the N-terminal portion of the protein [36]. The sfTSLP is human-specific, as there are no reports of a similar variant in other species [27]. sfTSLP mRNA is constitutively expressed in bronchial [34] and intestinal epithelial cells [37,38], fibroblasts [39], macrophages [17], and keratinocytes [40,41] Inflammatory stimuli specifically upregulate lfTSLP mRNA but not sfTSLP in human bronchial epithelial cells [42] and macrophages [17]. Despite evidence of a dichotomy of the two isoforms of TSLP in humans, the in vivo and in vitro functions of sfTSLP in humans are still largely unclear [4,43].

Mast cells, widely distributed in almost all human tissues [44,45], are strategically located in different compartments of the human lung [31,46,47,48]. Human lung mast cells (HLMCs) are recognized as central effectors in different asthma phenotypes [31,47,49]. The secretory granules of human mast cells contain preformed mediators, including tryptase and chymase [50]. Historically, human mast cells were classified into two subsets based on their protease content [51]: mast cells expressing tryptase are referred to as MC_T_, whereas those containing tryptase and chymase are known as MC_TC_ [51]. MC_TC_ are predominant in the lungs of patients with asthma [52,53,54]. Both proteolytic enzymes account for more than 25% of the total cellular protein [55,56]. Activated mast cells release tryptase and chymase [51,57,58], which have marked effects on the humoral and cellular components of the extracellular environment [50,59]. Previous studies have shown that tryptase and chymase can cleave several cytokines [59,60], promoting their activation [61,62,63,64,65,66]. Conversely, in other settings, mast cell proteases can exhibit anti-inflammatory activities by degrading proinflammatory cytokines [67,68]. Recent studies have shown that TSLP could also be a substrate for mast cell proteases. Prolonged incubation of nasal polyp extracts cleaves TSLP [69]. Moreover, it was demonstrated that TSLP can be cleaved by tryptase, although the cleavage site was not identified [60,69]. Finally, chymase caused only minor cleavage of TSLP [70].

Angiogenesis, the formation of new blood vessels, is fundamental to provide blood vessels to maintain tissue homeostasis [71,72]. In bronchial asthma, inflammatory angiogenesis is a critical factor in developing and sustaining airway remodeling [73,74]. Vascular endothelial growth factor-A (VEGF-A), released by several immune cells (e.g., macrophages, mast cells, basophils, and eosinophils) [57,75,76,77], is the most potent angiogenic factor.

In this study, we used strictly controlled conditions, as well as short incubation times and low enzyme/substrate ratios, to evaluate the early proteolytic events of recombinant human (rh) tryptase and chymase on TSLP by mass spectrometric analysis. We also investigated whether the TSLP and its fragments generated by enzymatic activity of tryptase and chymase can induce the release of angiogenic factors from human lung macrophages.

## 2. Results

### 2.1. Effects of Tryptase and Chymase on TSLP

**Figure 1:**
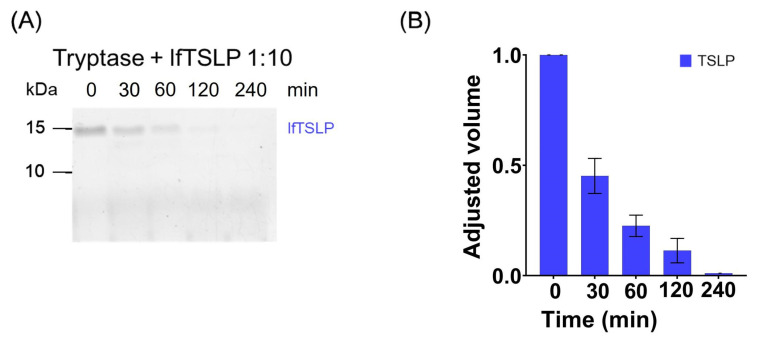
(**A**) Recombinant human lfTSLP (5 μg) was treated with recombinant human tryptase (0.5 μg) at 37 °C in the presence of heparin (1:10). Samples were withdrawn at 0, 30, 60, 120, and 240 min, and inactivated by heating for 10 min at 99 °C to stop the cleavage reaction. Each digestion mixture corresponding to 1 μg of protein was separated on 16% Tris-Tricine gel. The gel was stained with a colloidal Coomassie Brilliant Blue solution. (**B**) The reduction of the band intensity at ~15 kDa was quantified by densitometric analysis. The results show the mean ± SD of three independent experiments.

Mast cells are strategically located in different lung compartments of asthmatic patients [46,47,48,49,75]. These cells reside in close proximity to HLMs, which are the predominant immune cells in the human lung [48,78,79,80]. Activated HLMCs release tryptase and chymase [50,58]. This observation prompted us to investigate whether these proteases could potentially cleave TSLP. Recombinant human TSLP (≃15 kDa) was incubated with either tryptase or chymase at a 1:10 enzyme:substrate ratio by performing kinetic experiments (from 0 to 240 min) at 37 °C, and the digestion products were examined by SDS-PAGE. The reactions with tryptase were carried out in PBS in the presence of heparin [81], using a tryptase:heparin ratio of 1:10. Figure 1A shows that incubation of TSLP (~15 kDa) with tryptase resulted in a progressive decrease of the band corresponding to the intact protein. The densitometric analysis confirms that the intensity of the full-size TSLP band completely disappeared after 240 min of incubation (Figure 1B). These findings confirm and extend the previous observations [60,69] that TSLP is a substrate for tryptase.

**Figure 2:**
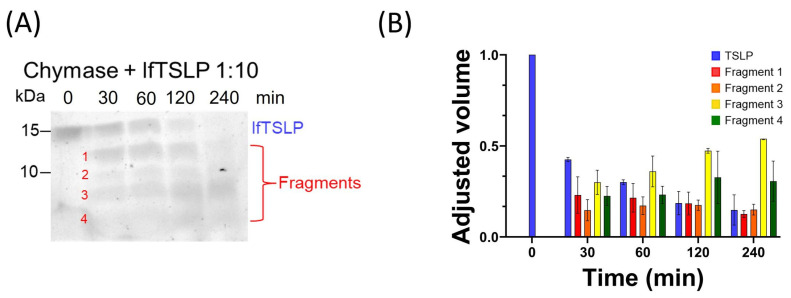
Cleavage analysis of lfTSLP by chymase. (**A**) Recombinant human TSLP (5 μg) was treated with chymase (0.5 μg at 37 °C). 1 μg aliquots were withdrawn at 0, 30, 60, 120, and 240 min, inactivated by heating for 10 min at 99 °C to stop the cleavage reaction and separated on 16% Tris-Tricine gel. The gel was stained with colloidal Coomassie Brilliant Blue solution. (**B**) Densitometric analysis of the cleavage products of TSLP generated by chymase (as shown in panel **A**). The progressive and marked reduction in the band intensity at ~15 kDa, and the appearance of several smaller fragments, indicated that TSLP is a substrate for chymase. The results show the mean ± SD of 3 independent experiments.

Similar experiments were performed to evaluate the effect of recombinant human chymase on TSLP. Figure 2A shows that the progressive incubation (from 0 to 240 min at 37 °C) of TSLP with chymase, using an enzyme:substrate ratio of 1:10, resulted in a progressive decrease of the band corresponding to the intact protein at 15 kDa. Several TSLP fragments were detected over a period of incubation of 30 to 240 min (Figure 2A). The densitometric analysis confirms that chymase efficiently degraded TSLP, generating several smaller fragments (Figure 2B).

### 2.2. Mass Spectrometry Analysis of Early Cleavage Products of TSLP Generated by Tryptase and Chymase

The recombinant TSLP was incubated with tryptase or chymase under controlled proteolytic conditions suitable for assessing a single cleavage event on the intact molecule by mass spectrometry. Preliminary experiments were performed for each protease to set up the optimal proteolysis conditions by mass spectrometry [82]. Supplementary Figure S1 shows the MALDI-MS spectrum of TSLP under non-proteolytic conditions. The mass signals recorded in the spectrum correspond to the mono (*m/z* 15,056.47), doubly (*m/z* 7528.7) and triply charged (*m/z* 5019.88) ions of intact TSLP, respectively, in agreement with the expected values (*m/z* 15,056.46, 7528.73, and 5019.49, respectively).

**Figure 3:**
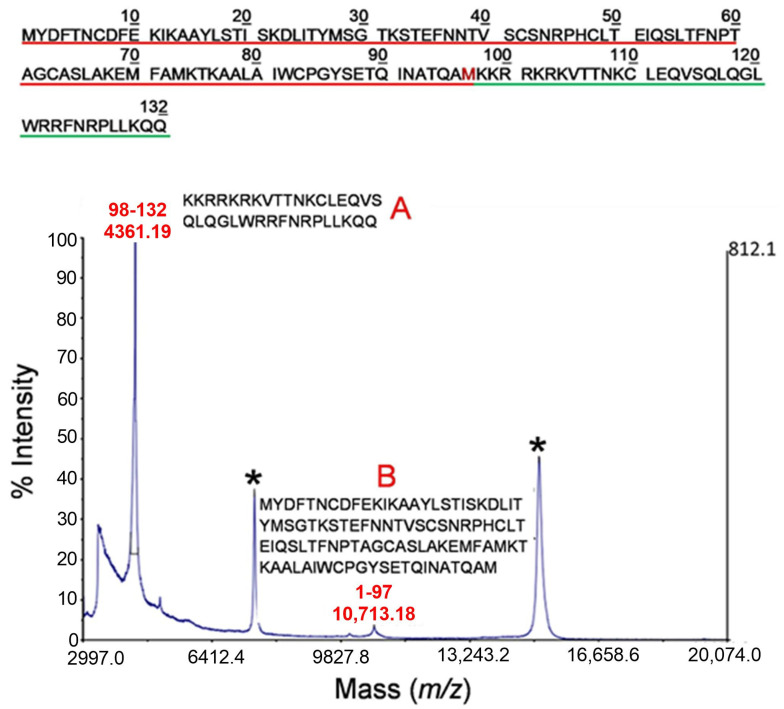
MALDI-MS analysis of TSLP following incubation with tryptase under strictly controlled conditions (E:S 1:1000 for 30 min at 37 °C). The signals marked with an asterisk correspond to the mono and doubly charged ions of the intact protein. Peaks at *m/z* 4361.19 and *m/z* 10,713.19 were assigned to the complementary peptides 98-132 and 1-97, respectively (marked A and B in the figure) originating from a single proteolytic cleavage between the peptide bond Met97-Lys98. The amino acid sequences of the two peptides (A and B) are shown in the inset and are underlined in red (A) or in green (B) in the upper panel of the figure. The tryptase preferential cleavage site is shown in red.

The proteolytic products of TSLP generated by tryptase under controlled conditions were then analyzed by MALDI-MS. The corresponding mass spectrum is shown in Figure 3. Besides the mass signals assigned to the intact protein (marked with an asterisk in the figure), two additional peaks were recorded in the spectrum. Based on their measured mass values and the TSLP sequence, the signal at *m/z* 4361.19 was assigned to the 98-132 fragment (theoretical *m/z* 4367.20) (indicated as A and the sequence of amino acids underlined in green in Figure 3); the peak at *m/z* 10,713.18 was identified as the complementary portion of the TSLP protein (fragment 1-97, theoretical *m/z* 10,714.28, indicated as B and the sequence of amino acids underlined in red in Figure 3). These results suggest that tryptase exerts an early proteolytic activity at the cleavage site located between the peptide bond Met97-Lys98.

**Figure 4:**
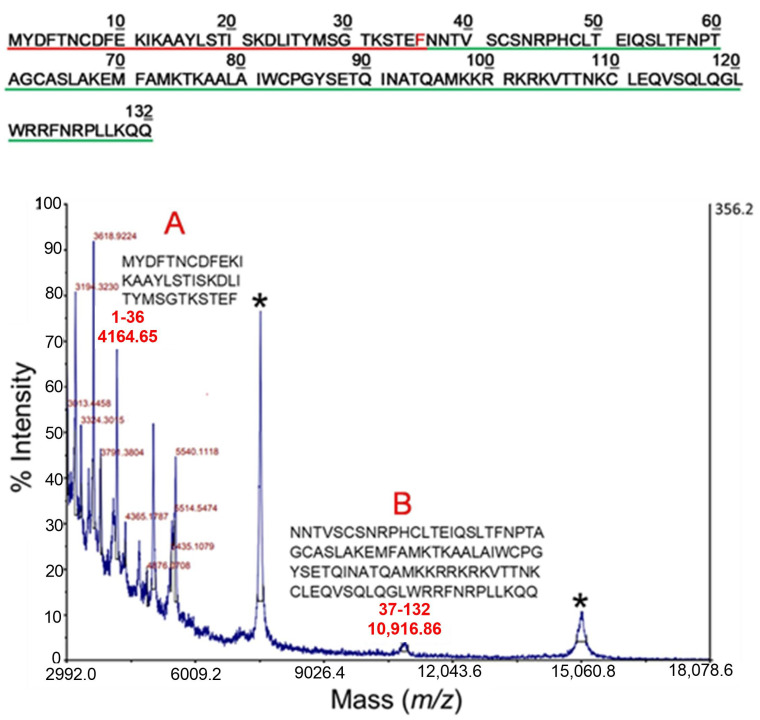
MALDI-MS analysis of TSLP following incubation with chymase under strictly controlled conditions (E:S 1:100 for 30 min at 37 °C). The signals marked with an asterisk correspond to the mono and doubly charged ions of the intact protein. Peaks at *m/z* 4164.65 and 10,916.86 were assigned to the complementary peptides 1-36 and 37-132, respectively (marked A and B in the figure) originating from a single proteolytic cleavage at Phe36. The amino acid sequences of the two peptides (A and B) are shown in the inset and are underlined in red (A) or in green (B) in the upper panel of the figure. The chymase preferential cleavage site between Phe36-Asn37 is shown in red. All other peaks in the spectrum were identified as sub-digestion products. Other fragments were observed at lower *m/z*, indicating further proteolytic cleavage of the two main fragments 1-36 and 37-132.

The proteolytic products of TSLP following incubation with chymase under limited proteolysis conditions were also analyzed by MALDI-MS. The corresponding mass spectrum is shown in Figure 4, where the peaks corresponding to the intact protein are marked with an asterisk. Among the other recorded signals, the peak at *m/z* 4164.65 (indicated as A in Figure 4) was assigned to the peptide 1-36 (theoretical molecular weight 4164.74 Da). The signal at *m/z* 10,916.86 (indicated as B in Figure 4) was associated to the complementary fragment 37-132 within the TSLP sequence (theoretic molecular weight 10,916.74 Da). These peptides were originated from a chymase preferential proteolytic site [59,83] located between the peptide bond Phe36-Asn37. All the other peaks in the spectrum were generated by sub-digestion of the main fragments.

### 2.3. Localization of the Early Cleavage Sites on the Three-Dimensional (3D) Structure of TSLP

**Figure 5:**
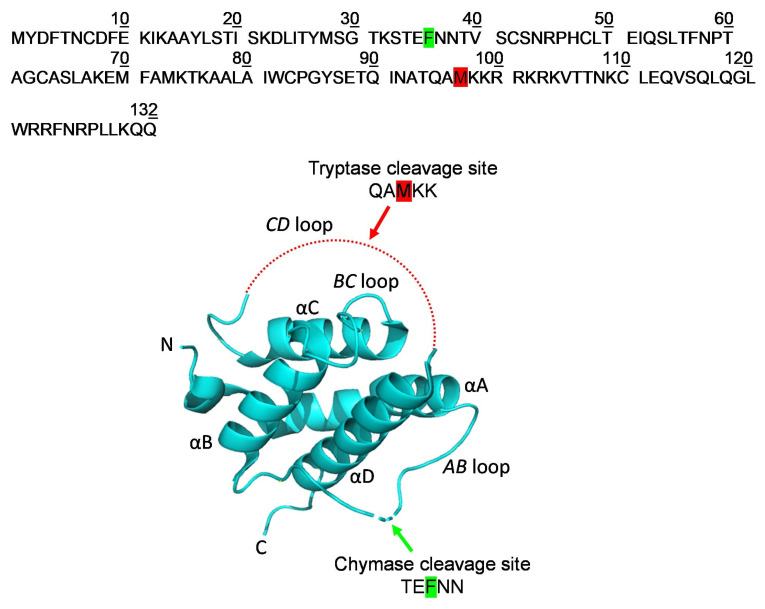
Tryptase and chymase preferential cleavage sites in the three-dimensional (3D) structure of TSLP. Upper panel: amino acid sequence of the mature form of TSLP. The tryptase and chymase specific proteolytic sites (Met97 and Phe36) are highlighted in red and green, respectively. Lower panel: ribbon representation of TSLP 3D structure according to Verstraete et al. [86]. The tryptase and chymase preferential cleavage sites are located within the *CD* and *AB* loops, highlighted in red and green, respectively. This representation provides insights into the spatial arrangement of the proteolytic sites within the mature form of TSLP.

Limited proteolysis experiments in combination with mass spectrometry represents a strategy to investigate the protein regions that are solvent-exposed and/or flexible enough to be accessible to proteases’ catalytic sites by identifying the early proteolytic events [82,84,85]. Tryptase rapidly cleaves TSLP in correspondence to the peptide bond Met97-Lys98, close to the C-terminus of the protein, whereas chymase specifically recognizes Phe36, located near the N-terminus of TSLP. Verstraete and collaborators reported that TSLP adopts a four-helix bundle with ‘up-up-down-down’ topology stabilized by three disulfide bridges (Cys34-Cys110, Cys69-Cys75, and Cys90-Cys137), in which the four α-helices—designated αA, αB, αC, and αD—are threaded via a *BC* loop and two long overhand *AB* and *CD* loop regions, with the latter largely invisible in the electron density maps [86]. According to their experimental data, Met97-Lys98 is located within the *CD* loop, a very flexible and not ordered region, which allows Met97 to be easily accessible to the tryptase catalytic site in controlled proteolysis conditions (Figure 5). Similarly, Phe36, the chymase preferential cleavage site, is located within the *AB* loop linking α helices A and B (Figure 5). Both regions (*CD* and *AB* loops) are endowed with a high degree of conformational flexibility that allowed tryptase and chymase to cleave specific peptide bonds.

### 2.4. Effects of TSLP and TSLP Fragments Generated by Tryptase and Chymase on Mediator Release from Human Lung Macrophages (HLMs)

**Figure 6:**
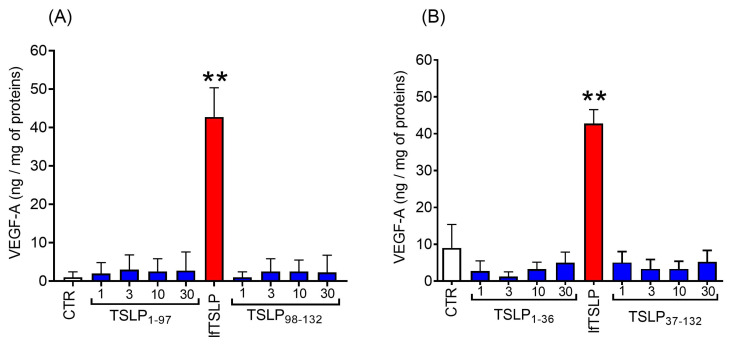
Effects of lfTSLP and TSLP fragments generated by tryptase and chymase on mediator release from human lung macrophages (HLMs). (**A**) Effects of lfTSLP (30 ng/mL) (red bar) and of increasing concentrations of the two TSLP fragments generated by tryptase (TSLP_1-97_ and TSLP_98-132_) (blue bars) and (**B**) of the two fragments generated by chymase (TSLP_1-36_ and TSLP_37-132_) (blue bars) on VEGF-A release from HLMs. The results show the mean ± SD of eight independent experiments performed with highly purified (≥99%) HLMs from different donors. ** *p* < 0.01 compared to control (CTR).

An important question to address was whether the early TSLP fragments generated by tryptase and chymase possess a bioactivity comparable to the native TSLP. We compared the effects of lfTSLP and the main cleavage products generated by tryptase and chymase on the release of angiogenic factors from HLMs. Figure 6A shows that lfTSLP (30 ng/mL) induced VEGF-A release from HLMs, whereas increasing concentrations (1–30 ng/mL) of the two TSLP fragments generated by tryptase, TSLP_1-97_ and TSLP_98-132_, had no effect on VEGF-A release from HLMs. Similarly, the two main TSLP fragments generated by chymase, TSLP_1-36_ and TSLP_37-132_, did not induce VEGF-A release from HLMs (Figure 6B).

### 2.5. Effect of PCSK3 on the Cleavage of TSLP

It has been reported that long-term (24 h) incubation of recombinant PCSK3, also known as furin, with TSLP generated two peptides (TSLP_1-103_ and TSLP_104-132_) without affecting the disulfide bonds of the protein [69]. PCSK3-treated TSLP induced the production of CCL17 from mDCs [69].

**Figure 7:**
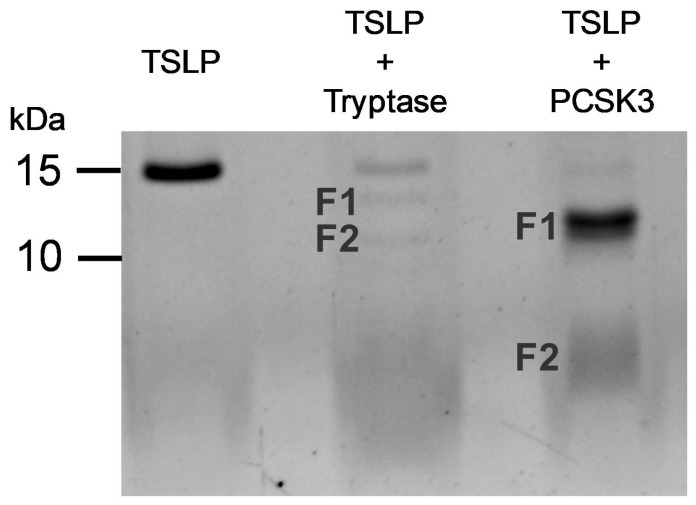
Cleavage analysis of TSLP by tryptase and PCSK3. Recombinant human non-glycosylated TSLP (2 μg) was incubated with tryptase (0.2 μg at 37 °C) for 1 h or with PCSK3 (0.88 μg at 37 °C) for 24 h at 37 °C. Aliquots were inactivated by heating for 10 min at 99 °C to stop the cleavage reaction and separated on 16.5% Tris-Tricine gel. The gel was stained with a colloidal Coomassie Brilliant Blue solution.

**Figure 8:**
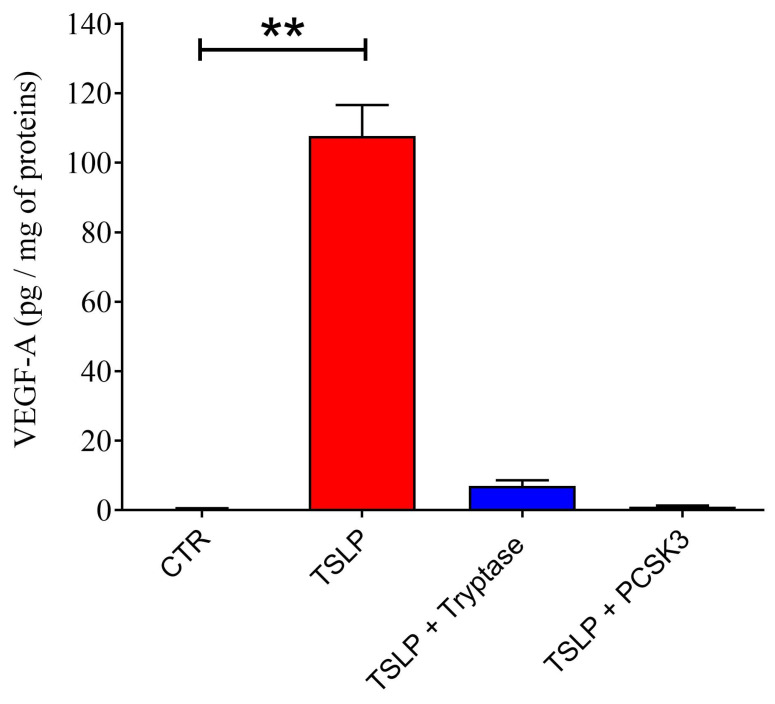
Effects of TSLP cleavage products generated by PCSK3 and tryptase on the release of VEGF-A from human lung macrophages (HLMs). Recombinant human non-glycosylated TSLP (2 μg) was incubated with tryptase (0.2 μg at 37 °C) for 1 h or with PCSK3 (0.88 μg at 37 °C) for 24 h at 37 °C. At the end of the incubation, aliquots of untreated TSLP, tryptase-treated TSLP, and PCSK3-treated TSLP were incubated (18 h, 37 °C) with HLMs in triplicate. At the end of the incubation, the supernatants were collected and VEGF-A concentrations were evaluated by ELISA. The results show the mean ± SD of a typical experiment out of three. ** *p* < 0.01.

Figure 7 shows that tryptase, after 1 h of incubation, partially cleaved TSLP inducing the formation of several smaller peptides (F1 and F2). Moreover, we confirmed the findings of Poposki and collaborators showing that long-term incubation of TSLP with PCSK3 completely cleaved TSLP generating two peptides of approximately 12 and 4 kDa (Figure 7). In parallel experiments, we compared the biological activity of products derived from TSLP upon treatment with PCSK3, tryptase, and untreated TSLP on HLMs. Figure 8 illustrates the results of a typical experiment showing that TSLP induced the release of VEGF-A from HLMs. By contrast, PCSK3-treated TSLP did not induce the release of VEGF-A from HLMs. Similarly, tryptase-treated TSLP caused a marginal increase in the release of VEGF-A from HLMs.

The results are representative of three independent experiments.

## 3. Discussion

We investigated whether TSLP may be a substrate for mast cell proteases (i.e., tryptase and chymase) released by HLMCs. The cleavage of recombinant lfTSLP by tryptase and chymase was studied in vitro by a limited proteolytic approach and the digestion products were identified by MALDI-MS. Our results showed that tryptase in controlled conditions cleaved TSLP, generating two major fragments corresponding to TSLP_1-97_ and TSLP_98-132_. In parallel experiments, the MALDI-MS results indicate a chymase site located at Phe36 generating TSLP_1-36_ and TSLP_37-132_. These findings demonstrate that the proteolytic activities of two mast cell-derived enzymes are directed against different sites. Tryptase cleaves TSLP in correspondence to the peptide bond Met97-Lys98, within the *CD* loop connecting the C and D α helices, close to the C-terminus of the protein. Chymase cleaves TSLP at the peptide bond Phe36-Asn37, placed within the *AB* loop linking α helices A and B, and located on the other side of the protein. Both regions are endowed with a high degree of conformational flexibility that allowed the proteases to easily cleave the adjacent peptide bonds.

Previous studies have demonstrated that mast cell-derived proteases can cleave several alarmins. In particular, chymase has been shown to cleave HMGB1 and IL-33 [87,88]. A detailed study reported that chymase cleaved several cytokines but caused only minor cleavage of TSLP as observed by using SDS-PAGE [70]. More recently, the same research group presented evidence that TSLP is a substrate for tryptase, although the cleavage site was not identified [60]. Poposki and collaborators reported that prolonged (24 h) incubation of TSLP with mast cell proteases, including tryptase, chymase, and cathepsin G led to TSLP digestion, as assessed by Western blot [69]. In our experiments, we used strictly controlled conditions, including a short time of incubation and a relatively low enzyme/substrate ratio, to mimic in vivo conditions and evaluate the physiological significance of the tryptase and/or chymase proteolytic activity on TSLP. Reports showing cleavage under more prolonged [69] or excessive conditions are clearly less relevant when considering the in vivo scenario. Attempts to demonstrate substrate cleavage after 10 to 24 h are unlikely to be biologically meaningful [70] since other proteases and protease inhibitors in vivo would likely inactivate the enzyme within minutes or few hours. Thus, in vitro cleavage analysis for extended periods is probably not biologically relevant. A similar incubation time of chymase with VIP was appropriately taken in a previous study [89].

An important aspect to consider is the presence of several proteases (e.g., tryptase, chymase, and carboxypeptidase A3) in the mast cell granules [50,90]. These proteases are presumably released together [56], which could impact the cumulative effects on target molecules. The initial cleavage by one enzyme could potentially alter the structure and make the target molecule more susceptible to proteolysis by other enzymes. To better understand the TSLP sensitivity to cleavage by mast cell proteases, further studies investigating the combined effects of multiple proteases should be carried out.

A relevant question to address was whether tryptase and chymase could alter the bioactivity of TSLP. To this end, we compared the effects of TSLP and the main cleavage fragments generated by tryptase and chymase on HLM activation. The proteolytic activity of tryptase can lead to three completely different biological effects [91]. This enzyme can cleave the proteinase-activated receptor 2 (PAR-2), inducing its activation [92], and it can also cleave the EGF-Like Module-Containing Mucin-Like Hormone Receptor-Like 2 (EMR2) subunit α (EMR2α), weakening the association of EMR2α/EMR2β to potentiate vibration-dependent mast cell degranulation [93]. Alternatively, tryptase can cleave IL-33, potentiating its bioactivity [63]. Finally, this protease can degrade the neuropeptide vasoactive intestinal peptide (VIP) [94,95] and counteract the smooth muscle relaxant effect of VIP [96]. On the other hand, there is evidence that chymase can also modulate the biological activity of several cytokines. In particular, this chymotrypsin-like enzyme can activate TGF-β [61,62], IL-33 [63,87,88], stem cell factor (SCF) [64], IL-1β [65], and IL-18 [66]. By contrast, chymase can inactivate TNF-α [67], IL-6 [68], and IL-13 [68].

It has been demonstrated that prolonged incubation of nasal polyp (NP) extracts with TSLP generated two main fragments corresponding to TSLP_1-97_ and TSLP_98-132_, which remained linked through disulfide bonds as a dimerized form [69]. Although the synthetic peptides and their mixture did not induce the production of CCL17 from peripheral blood mononuclear cells (PBMCs), TSLP peptides generated by furin (PCSK3) were dimerized through a disulfide bond and induced CCL17 from mDCs [69]. We have confirmed the findings by Poposki and collaborators, showing that long-term (24 h) incubation of TSLP with furin generated two peptides of approximately 12 and 4 kDa. In our experimental model, the TSLP peptides generated by long-term incubation with PCSK3 did not induce VEGF-A release from HLMs. The different experimental system for detecting the biological effects of TSLP and its fragments could explain these latter differences. We cannot exclude the possibility that TSLP peptides generated in vivo by tryptase, chymase and other proteolytic enzymes might remain linked through disulfide bonds as a dimerized form and could activate HLMs.

Mast cells are strategically located in different compartments of the lung in asthmatic patients [47,49], and are canonically viewed as central effectors in different asthma phenotypes [31,97]. In particular, mast cells are implicated in early and late inflammatory responses in bronchial asthma [97,98]. Moreover, there is evidence that mast cells and their mediators play a critical role in several aspects of airway remodeling in asthma [31]. The above considerations have led to the development of biological therapies targeting mast cells or their receptors/mediators for the treatment of severe asthma [31]. There is overwhelming evidence that TSLP is a master orchestrator of the immune response in asthma pathobiology [5,32,33,43]. Our results unveil an intricate interplay between mast cell-derived proteases and TSLP with possible implications in asthma pathobiology.

## 4. Materials and Methods

### 4.1. Reagents

The following were purchased: recombinant human TSLP (SRP4896, Sigma-Aldrich, St. Louis, MO, USA and BT-NBP2-35083, Novus Biologicals, Centennial, CO, USA) expressed in *E. coli* (protein without glycosylation), recombinant human β-tryptase (G563A, Promega Biotech, Madison, WI, USA), and recombinant human chymase (S-C8118, Merck Life Science, Milan, Italy). TSLP_1-36_, TSLP_37-132_, TSLP_1-97_, and TSLP_98-132_ were synthetized by ProteoGenix SAS (Schiltigheim, France) and their purity was >98% evaluated by mass spectrometry. Recombinant human furin (PCSK3) (1503-SE, R&D System, Minneapolis, MN, USA), bovine serum albumin, L-glutamine, antibiotic–antimycotic solution (10,000 IU/mL penicillin, 10 mg/mL streptomycin, and 25 μg/mL amphotericin B), RPMI 1640, fetal calf serum (FCS) (endotoxin level < 0.1 EU/mL), 1,4-Piperazinediethanesulfonic acid (PIPES), PBS (14200067, Gibco^TM^, ThermoFisher Scientific, Waltham, MA, USA), Percoll^®^ and Triton X-100 (Sigma-Aldrich, St. Louis, MO, USA), detoxified lipopolysaccharide (LPS) (from *E. coli* serotype 0111:B4), IL-4 (Miltenyi Biotec, Bologna, Italy), heparin (PharmaTex Italia, Milan, Italy), and rabbit polyclonal antibody anti-human TSLP (ab109229, Abcam, Milan, Italy) were also obtained.

### 4.2. In Vitro TSLP Proteolysis by Tryptase, Chymase and PCSK3

Recombinant human non-glycosylated TSLP expressed in *E. coli* (BT-NBP2-35083, Novus Biologicals, Milan, Italy or SRP4896, Sigma-Aldrich, St. Louis, MO, USA) was treated with recombinant human β-tryptase (G563A, Promega Biotech, Madison, WI, USA) or chymase (S-C8118, Sigma-Aldrich, Milan, Italy) in limited proteolysis conditions. The reactions with tryptase or chymase were carried out in PBS using an enzyme:substrate ratio of 1:10. For each condition, the reactions were performed at different times at 37 °C. The hydrolysis with tryptase was carried out in the presence of a tryptase:heparin ratio 1:10 [81]. Tryptase, a tetrameric serine protease, in which the active sites face a narrow central pore, is stabilized by interaction with heparin [81]. The reactions were examined for different time intervals (from 0 to 240 min) and stopped by heating for 10 min at 99˚C. In other experiments, we evaluated the cleavage of TSLP by tryptase and PCSK3 (furin). Recombinant human TSLP (SRP4896, Sigma-Aldrich) (2 μg) was treated with tryptase (0.2 μg at 37 °C) for 1 h or with PCSK3 (0.88 μg at 37 °C) for 24 h at 37 °C. In all experiments, the reaction products were separated on 16.5% Tris-Tricine gels and visualized by staining with colloidal Coomassie Brilliant Blue.

### 4.3. Limited Proteolysis and MALDI-MS Analysis

Recombinant human TSLP (SRP4896, Sigma-Aldrich) was treated with β-tryptase (G563A, Promega) or chymase (S-C8118, Sigma-Aldrich) in limited proteolysis conditions and analyzed by Matrix Assisted Laser Desorption/Ionization-Mass spectrometry (MALDI-MS) in linear mode. The reactions were carried out on 1 μg of TSLP. Tryptase was added with an enzyme:substrate ratio of 1:1000 (*w*/*w*) for 30 min, while chymase was used at an enzyme:substrate ratio of 1:100 (*w*/*w*) for 30 min. These experimental conditions were set up based on optimal hydrolysis conditions for each protease [82]. For MALDI-MS analyses, 0.5 μL of each peptide mixture was mixed with an equal volume of α-cyano-4-hydroxycinnamic acid as matrix (10 mg/mL) in 0.2% trifluoroacetic acid (TFA) in 70% acetonitrile, loaded onto the metallic sample plate, and air-dried. The peptide mixture was analyzed in linear mode by a 4800 plus MALDI TOF-TOF mass spectrometer (AB SCIEX, Toronto, ON, Canada) and using the 4000 Series Explorer (TM) software (AB SCIEX, Toronto, ON, Canada) (version 3.5) to detect the released fragments, in order to identify the cleavage sites on TSLP. Mass calibration was performed using the MH^+^ and MH_2_^2+^ ions of a protein mixture containing insulin and apomyoglobin to ensure accurate mass determination and calibration [99].

### 4.4. Localization of the Early Cleavage Sites on the Three-Dimensional (3D) Structure of TSLP

The localization of the early cleavage sites on the three-dimensional (3D) structure of TSLP was determined using the 3D structure described by Verstraete and collaborators [86]. The PyMOL software (DeLano Scientific LLC, Palo Alto, CA, USA) (2.5.4 version) was used to visualize the 3D structure of the TSLP protein and to explore potential cleavage sites for tryptase and chymase [100].

### 4.5. Isolation and Purification of Human Lung Macrophages (HLMs)

The study protocol was approved by the Ethics Committee of the University of Naples Federico II (Prot. 09/22 of 4 August 2022), and informed consent was obtained from donors. Macrophages were isolated and purified from macroscopically normal lung tissue obtained from 27 patients (age range: 60–81 years) affected by lung adenocarcinoma undergoing lobectomy [101]. Patients included in the study were negative for hepatitis C virus (HCV), hepatitis B surface Ag (HBsAg) and HIV-1 infections. None of the patients had received chemotherapy or radiotherapy prior to surgery. Freshly resected lung tissue was obtained intraoperatively and finely minced with scissors. The minced tissue was then extensively washed with PIPES buffer over Nytex cloth (120 μm pore size) (Tetko Elmsford, NY, USA). After Percoll gradient centrifugation, the cells were suspended (10^6^ cells/mL) in RPMI 1640 with 5% FCS, 2 mM L-glutamine, and 1% antibiotic–antimycotic solution and incubated at 22 °C in 24-well plates (Falcon, Becton Dickinson, Milan, Italy). After 12 h, the medium was removed, and the plates were gently washed with RPMI 1640. More than 99% of adherent cells were macrophages, as evaluated by flow-cytometric analysis [102].

### 4.6. Cell Incubations

HLMs were cultured in 24-well plates in RPMI 1640 medium supplemented with 5% FCS, 2 mM l-glutamine, and 1% antibiotic–antimycotic solution, as previously described [17]. HLMs were treated with TSLP and its proteolytic fragments for 16 h at 37 °C. At the end of incubations, the supernatants were collected and stored at −80 °C for subsequent ELISA quantification of VEGF-A. Cell lysis in the plates was carried out using 0.1% Triton X-100 for total protein quantification by a Bradford-based assay (Bio-Rad, Segrate, Milan, Italy).

### 4.7. ELISA Assays

Cytokine concentrations were measured using commercially available ELISA kits for VEGF-A (31.3–2000 pg/mL) (R&D System, Minneapolis, MN, USA). The number of adherent macrophages varies among wells and different experiments; therefore, the results were normalized for the total protein content in each well, determined in the cell lysates (0.1% Triton X-100) by the Bradford-based assay. The normalized cytokine release was expressed as pg of specific cytokine/mg of total proteins.

### 4.8. Statistical Analysis

Statistical analysis was performed by Prism 9 (GraphPad Software, San Diego, CA, USA). The data are expressed as mean values ± standard deviation (SD) of the indicated number of experiments. Statistical comparisons were performed by Student’s *t*-test or one-way analysis of variance (ANOVA) followed by Dunnett’s test (when a comparison was made against a control) or Bonferroni’s test (when a comparison was made between each pair of groups). Values of *p* < 0.05 were considered statistically significant.

## Data Availability

The original contributions presented in the study are included in the article/Supplementary Material. Further enquiries can be directed to the corresponding authors.

## Supplementary Materials

The following supporting information can be downloaded at: https://www.mdpi.com/article/10.3390/ijms25074049/s1.

## Abbreviations

CTR: control; DC, dendritic cell; EGF-Like Module-Containing Mucin-Like Hormone Receptor-Like 2 (EMR2) subunit α (EMR2α); FCS, fetal calf serum; HBsAg, hepatitis B surface Ag; HCV, hepatitis C virus; HEK 293 cells, human embryonic kidney 293 cells; HLM, human lung macrophage; HLMC, human lung mast cell; IL-7Rα, interleukin 7 receptor-α; JAK, Janus kinase; lfTSLP, long form TSLP; LPS, lipopolysaccharide; MALDI-MS: Matrix Assisted Laser Desorption Ionization-Mass Spectrometry; MC_T;_ mast cell tryptase^+^; MC_TC_, mast cell tryptase^+^ chymase^+^; mDC, myeloid dendritic cell; NP, nasal polyps; PBMC, peripheral blood mononuclear cell; PBS, phosphate buffer saline; PCSK3, propotein convertase subtilisin/kexin 3; rh, recombinant human; PIPES, 1,4-Piperazinediethanesulfonic acid; SCF, stem cell factor; SD, standard deviation; sfTSLP, short form TSLP; STAT5, signal transducer and activator of transcription 5; TFA, trifluoroacetic acid; TSLP, thymic stromal lymphopoietin; TSLPR, thymic stromal lymphopoietin receptor; VEGF-A, vascular endothelial growth factor-A; VIP, vasoactive intestinal peptide; 3D, three-dimensional.
