# Supplementary material for: Thymic Stromal Lymphopoietin (TSLP) Is Cleaved by Human Mast Cell Tryptase and Chymase
Source: Int J Mol Sci. 2024 Apr 5;25(7):4049. doi: 10.3390/ijms25074049 (PMC11012384; doi:10.3390/ijms25074049)
Supplement: Supplementary file 1 [file ijms-25-04049-s001.zip › ijms-2907178-supplementary.pdf]

International Journal of *Molecular Sciences*

MDPI

Supplementary Material: Thymic Stromal Lymphopoietin (TSLP) Is Cleaved by Human Mast Cell Tryptase and Chymase

Luisa Canè [1,2,3,†], Remo Poto [1,2,†], Francesco Palestra [1,2], Ilaria Iacobucci [3,4], Marinella Pirozzi [5], Seetharaman Parashuraman [5], Anne Lise Ferrara [1,2], Amalia Illiano [6], Antonello La Rocca [6], Edoardo Mercadante [6], Piero Pucci [3], Gianni Marone [1,2,5,7], Giuseppe Spadaro [1,2,7], Stefania Loffredo [1,2,5,7], Maria Monti [3,4,*] and Gilda Varricchi [1,2,5,7,*]

[1] Department of Translational Medical Sciences, University of Naples Federico II, 80131 Naples, Italy; canel@ceinge.unina.it (L.C.); remo.poto@gmail.com (R.P.); f.palestra97@gmail.com (F.P.); anneliseferrara@gmail.com (A.L.F.); marone@unina.it (G.M.); spadaro@unina.it (G.S.); stefania.loffredo2@unina.it (S.L.)
[2] World Allergy Organization (WAO), Center of Excellence (CoE), 80131 Naples, Italy
[3] CEINGE Advanced Biotechnologies F. Salvatore, 80131 Naples, Italy; ilaria.iacobucci@unina.it (I.I.); pucci@unina.it (P.P.)
[4] Department of Chemical Sciences, University of Naples Federico II, 80126 Naples, Italy
[5] Institute of Experimental Endocrinology and Oncology, National Research Council (CNR), 80131 Naples, Italy; m.pirozzi@ieos.cnr.it (M.P.); seetharaman.parashuraman@cnr.it (S.P.)
[6] Thoracic Surgery Unit—Istituto Nazionale Tumori IRCCS Fondazione G. Pascale, 80131 Naples, Italy; a.illiano@istitutotumori.na.it (A.I.); a.larocca@istitutotumori.na.it (A.L.R.); edoardo.mercadante@istitutotumori.na.it (E.M.)
[7] Center for Basic and Clinical Immunology Research (CISI), University of Naples Federico II, 80131 Naples, Italy
* Correspondence: montimar@unina.it (M.M.); gildanet@gmail.com (G.V.)
† These authors contributed equally to this work.

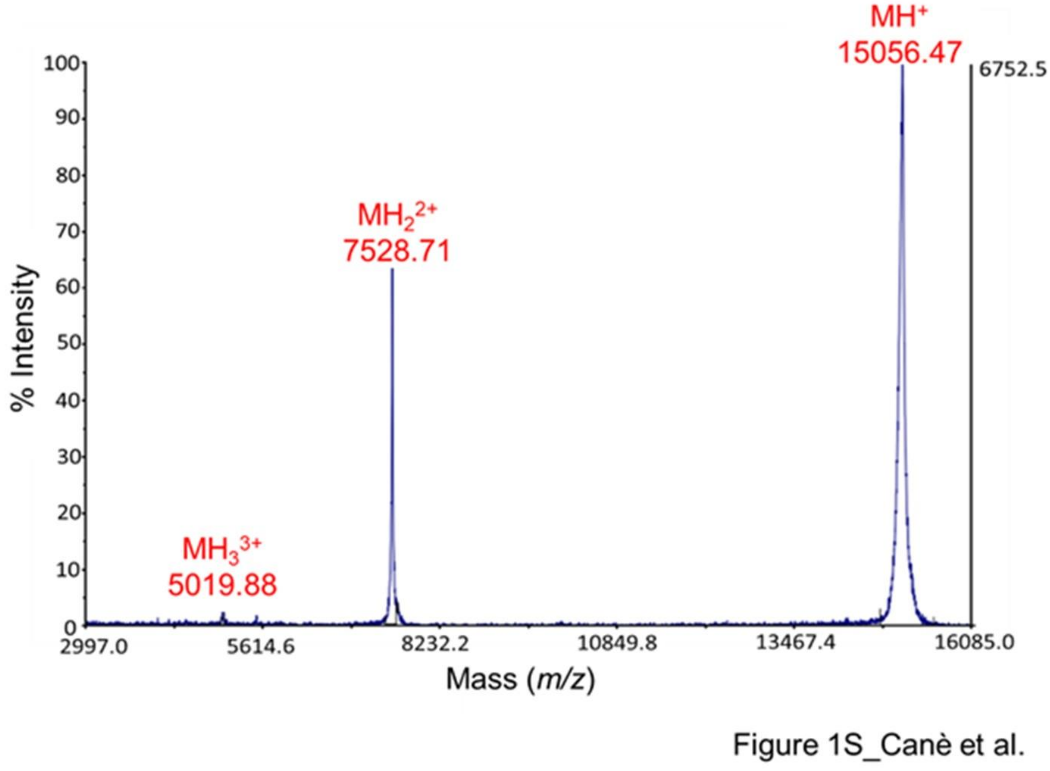

Figure S1. MALDI-MS spectra of recombinant human TSLP in linear mode. The mass signals at m/z 15056.47, 7528.71, and 5019.88 correspond to the mono-, doubly- and triply-charged ions of the intact protein, respectively. From the measured mass value of these ions, a molecular weight of 15056.18 for TSLP could be calculated in agreement with the expected value (15056.46 Da).
